# Good News or Bad News?

**DOI:** 10.31662/jmaj.2024-0025

**Published:** 2024-03-18

**Authors:** Kunihiko Matsui

**Affiliations:** 1 Department of General Medicine and Primary Care, Kumamoto University Hospital, Kumamoto, Japan

**Keywords:** COVID-19, Japanese, home death

The recent worldwide pandemic of coronavirus infection disease 2019 (COVID-19) has perished many people and changed our daily lives. During this period, various problems beyond the direct medical care were raised not only by legal regulations, including behavioral restrictions to prevent the spread of infection, but also by forced new lifestyles, such as wearing masks and vaccinations as social manners. In addition, in end-of-life (EOL) care, where to meet the EOL is a big issue more than ever.

Various factors may influence to fulfill the wishes and preferences of patients and their families for EOL care. In a recent survey of the Japanese in 2021, 17.2% of deaths occurred at home, while 65.9% occurred at hospitals ^(1)^. From a historical point of view, the percentage of people died at home was as much as 82.5% in 1951, and this rate had decreased consistently. It reached 12.2% in 2005 and has slightly increased every year ever since. Also, a recent survey showed that 43.8% of people wished for home death if their life expectancy was less than 1 year with a hypothetical scenario ^(2)^. The study by Sun et al. published in this issue of JMAJ showed an increase in home deaths in Japan during the recent COVID-19 pandemic and the factors associated with EOL care at home. Could this be interpreted as good news because of the patient and family’s wishes for EOL care at home being fulfilled even in the midst of the COVID-19 disaster? Also, this study showed that the high proportion of home death was related to the urban location and the sufficiency of health care and support delivery system ^(3)^. There are many possible interpretations of these results.

In Japan, even during the pandemic of COVID-19, deaths from COVID-19 were accounted for less than 1% of all deaths, and it is unrealistic to say that COVID-19 itself had a significant impact on the overall number of deaths, as the authors said. It is also worth remembering that at the time of the pandemic, it was reported that family members were unable to accompany with their patients’ EOL care. For the medical providers at the time, it was difficult to accommodate the requests for EOL care for the patient and his/her family under the rapid course of the disease. It is difficult to imagine that COVID-19 had a significant impact on the increase in EOL care at home.

In the early days of the outbreak of COVID-19, there was confusion in the medical care, which would have had an impact on the quality of care provided at that time. The disruption in the health care setting had a significant impact on the acceptance and provision of care for the patients including other than COVID-19. This may be owing to the bed availability and the inability to provide inpatient care as the authors referred. From the patient perspective, it is possible that behavioral restrictions during the pandemic of COVID-19 impeded access to outpatient care, changing patient behavior, and increased EOL care at home from diseases other than COVID-19. From these considerations, it is unlikely that wishes from the patient side influenced the increase in the proportion of home deaths during the COVID-19 pandemic.

Alternatively, how should we interpret that the rate of EOL care at home increased in urban areas? The regional disparity in EOL care at home has been pointed out. A previous report has shown that the rate of home deaths was higher in areas with a higher number of practitioners providing EOL care. This was not related to city size ^(4)^. The provision of a well-developed medical care and support system would be related to the availability of EOL care at home. It could be interpreted that the differences in the quality of medical care provided in urban and rural areas has reflected to the results.

From the above, it seems difficult to say that the proportion of home deaths increased during the pandemic of COVID-19 as a result of the provided high quality of care and the fulfillment of the patients’ and their families’ wishes. Also, this would not be the result of the COVID-19 pandemic boosting the provision of the desired EOL care at home. Rather, it would be due to changes in patients’ behavior owing to mobility restrictions. The decrease in inpatient death should be viewed as the effect of the restrictions from the COVID-19 pandemic and the effect of diseases other than COVID-19. This would have reflected the disruption of the healthcare delivery system, especially in the early stages of the pandemic.

This study is an ecology research; there should be caution to the results. As the authors state, it is not possible to show a causal relationship, and the results are subject to a variety of factors. The magnitude of the impact from COVID-19 itself is not known. In addition, it is possible that we are simply looking at a recent trend that has been increasing EOL care at home. How could we interpret the results? Were they good news that the patients and their families had received the EOL care they wanted at home, or bad news showed the variations in the quality of care provided? Although there are many possibilities, I believe the results of this study should be interpreted that the problems in community health care systems have become apparent and emphasized by this disaster. Will EOL care at home continue to increase more than ever after the end of the COVID-19 pandemic? We will have to wait and see what happens in the future. However, this study shows that the quality of provided medical care must be maintained and improved, regardless of whether it is in urban or rural areas under various unexpected circumstances.

## Article Information

### Conflicts of Interest

None

### Disclaimer

Kunihiko Matsui is one of the Editors of JMA Journal and on the journal’s Editorial Staff. He was not involved in the editorial evaluation or decision to accept this article for publication at all.

## References

[1] Health and Welfare Statistics 2022. Vol. 1: Population and Households; Chapter 2, Demographics [Internet]. Tokyo: Ministry of Health, Labor and Welfare; 2023 Apr [cited 2024 Feb 12]. Available from: https://www.mhlw.go.jp/toukei/youran/indexyk_1_2.html. Japanese.

[2] Report on the Survey of Attitudes toward Medical Treatment and Care in the Final Stage of Life. Survey on Attitudes toward Medical Treatment and Care in the Final Stage of Life [Internet]. Tokyo: Ministry of Health, Labor and Welfare; 2023 Dec [cited 2024 Feb 12]. Available from: https://www.mhlw.go.jp/toukei/list/dl/saisyuiryo_a_r04.pdf. Japanese.

[3] Sun Y, Iwagami M, Inokuchi R, et al. Change in the proportion of death at home during the COVID-19 pandemic and its associated factors in the municipality level: a nationwide study in Japan. JMA J. 2024;7(2):213-221. 10.31662/jmaj.2023-0165 PMC11074502 38721095

[4] Kikuchi J. Report on Ministry of Health and Labor Administration Promotion Research Project 2016: Regional correlation analysis of home death ratio [Internet]. Tokyo: Ministry of Health, Labor and Welfare; 2018 Nov 27 [cited 2024 Feb 12]. Available from: https://mhlw-grants.niph.go.jp/system/files/2017/171011/201701008B_upload/201701008B0004.pdf. Japanese.

